# The demonisation of psychiatrists in fiction (and why real psychiatrists might want to do something about it)

**DOI:** 10.1192/pb.bp.113.045633

**Published:** 2014-08

**Authors:** Jacqueline Hopson

**Affiliations:** 1 University of Sheffield

## Abstract

To encourage psychiatric practitioners to be aware of and to work to counteract the representations of the profession as evil manipulators in fiction, film and popular culture. A wide-ranging number of representative sources portraying psychiatrists are explored. It is demonstrated that psychiatry is overwhelmingly presented in a damagingly negative light.

Psychiatry has never enjoyed the respect and social prestige of other medical specialties. In a 2004 survey of medical students, Rajagopal *et al* found the following reasons were ‘prominent for not choosing psychiatry’: ‘boring, unscientific, depressing, stressful, frustrating and “did not enjoy rotation” (in that order)’.^1^ The World Psychiatric Association’s 2010 paper, ‘WPA guidance on how to combat stigmatization of psychiatry and psychiatrists’,^2^ is one of many papers, published over many years, which address the problem of psychiatry’s negative image. Psychiatric practitioners have been regarded with suspicion, fear and defensive ridicule by medical colleagues, as well as by the average person on the street. But something worse is happening in fiction, film and popular culture: the psychiatrist is a villain. He (for the fictional psychiatrist is almost invariably male) is depicted as a murderer, rapist, paedophile, charlatan or crook. At his most benevolent, he is an ineffectual fool, unable even to manage his own life. This is a problem for all of us. The damage done to real, competent professionals by these fictional representations is insignificant when compared with the impact on potential or current patients. Psychiatrists (and I use this as a cover-all term for psychoanalysts, therapists and psychologists for the purpose of this article) are frequently considered difficult to approach by the desperate and distressed people who might need their help, and the stigma associated with mental illness seems firmly established for patients already in the care of these apparently strange professionals who are perceived as meddling with our minds and who, if fictions are to be believed, often have appallingly evil tendencies. It is these fictional portrayals of psychiatrists that present the most prevalent and readily accessible images of the profession to the general public. These are the damagingly negative, widespread representations that dominate the way in which psychiatrists are seen outside the world of medicine. In this article, I shall refer to a number of texts which I have chosen because they are representative fictional depictions of psychiatrists and because they are well known; or because they are particularly shocking and stigmatising texts which have, in many cases, enjoyed wide popular consumption.

## Many psychiatrists see literature as valuable

Over the past 30 years or so, many doctors and psychiatrists have published articles advocating the study of literature and the arts, a significant number of such articles having been published in this journal. These writers appear to believe (quite rightly in my view) that wide reading in fiction gives practitioners access to a greater range of human experience, including that of being ill and also of being mentally ill. Such articles argue that studying the arts is likely to increase the empathy of medical and psychiatric practitioners. There are some writers who make a strong case for teaching textual analysis to doctors and it is clear that this could indeed be useful, with the emphasis of this approach on the selection of material, the way it is presented and the questioning of the reliability of the narrator. Crucially, textual analysis recognises that there is more than one way to tell a story, and that the patient’s own narrative is as important as the doctor’s case notes. The rise of narrative medicine and the increased number of medical humanities publications show that the arts are highly regarded by medicine and psychiatry. It seems these professions want to make use of the arts as helpful tools in understanding and treating patients. However, as far as psychiatry is concerned, this respect is not reciprocated. The arts present psychiatrists in a worryingly negative light.

## Psychiatry’s anti-heroes

**Fig 1:**
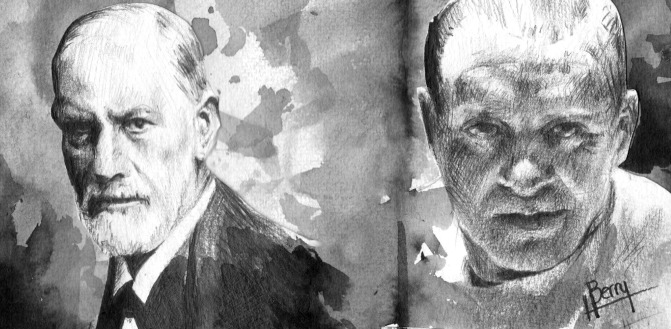
Healing doctor or evil torturer: Sigmund Freud and Hannibal Lecter, played by Anthony Hopkins in *The Silence of the Lambs*. Artist: Hester Berry.

Fiction’s devilish Hannibal Lecter from *The Silence of the Lambs*^3,4^ jostles with Sigmund Freud for first place as the most readily recognised psychiatrist in popular culture (Fig. 1). Lecter has qualities that many of us are afraid that psychiatrists possess. He can read people as easily as if they were books. By mere observation, he can deduce the smallest facts about those he sees. As consumers of the novel or film, we fearfully (and erroneously) deduce that psychiatrists can look into our minds and see straight through to our innermost secrets. As a result, prospective patients will see the psychiatrist as terrifying if they regard him not as a trustworthy ally, but a potential enemy. In popular culture, Hannibal Lecter is just one of many totally evil, very frightening, fictional psychiatrists.

There is a major group of villains in another popular format: comic books. Marvel Comics and DC Comics have been in wide circulation since the 1930s and popularised such favourites as Batman, Spiderman and Superman. As well as these superheroes, the comics also feature many perpetrators of evil who use their intellect, education and knowledge of human behaviour to attack others. These are the comic-book psychiatrists. Psychiatry appears to be one of the professions most heavily - and negatively - represented in comic books. There seems to be a shorthand code at work here: if you want a wily, powerfully evil villain, make him a psychiatrist. The Arkham Lunatic Asylum runs through many story lines in DC Comics. Arkham is a terrifying place, where, as sometimes happens in real psychiatric hospitals, inconvenient people are easily locked away without the benefit of a legal trial (e.g. Elizabeth Packard, who was locked up as ‘mad’ by her husband as she disagreed with his religious views^5^). The psychiatrist’s ability to legally detain those apparently in his power is a reasonable basis for terror in the real world. Comic-book asylum head, Jeremiah Arkham, becomes a patient in his own hospital, following the career of many fictional psychiatrists who go mad themselves. This is another problem for patients in the real world. Madness - the loss of ability to distinguish reality from hallucination, unreason from reason - is, perhaps, one of our greatest fears; and this madness is projected on to psychiatrists in literature and popular culture. We are afraid that these doctors must be ‘crazy’ themselves since they understand the mad. Erving Goffman’s notion of stigma by contagion is relevant here, the psychiatrist acquiring the social unacceptability of the mental patient.^6^

Other typical comic-book psychiatrists are Dr Voodoo (*Avenger of the Supernatural*, Marvel Comics, 2009), who obviously practices dark arts and who wears a string of shrunken heads around his waist - a clear reference to the derogatory term, ‘head-shrinker’; Dr Faustus (*Batman Gotham Knight*, DC Comics, 2000), whose traditional relationship with the devil is well known; and the demented Professor Hugo Strange (*Captain America*, Marvel Comics), whose name and appearance send a clear message to readers. These, along with characters such as Hannibal Lecter, are the fictional psychiatrists familiar to a wide audience. They are the well-known, albeit unreal, representatives of the profession which offers help to the vulnerable and distressed people with mental illness. Should we be surprised that patients are frightened to approach psychiatrists?

## Most shocking excesses

Novels, both popular and literary, frequently depict psychiatrists as evil. Fictional psychiatrists are commonly rapists, murderers and sexual abusers of the vulnerable, notable examples being found in Stieg Larsson’s, *The Girl With the Dragon Tattoo*,^7^ Hanif Kureishi’s *Something to Tell You*^8^ and Luke Rhinehart’s, *The Dice Man*,^9^ the last a tale of a string of appalling abuses of patients by a completely amoral doctor. *Electricity*, by Victoria Glendinning,^10^ has one of the most shocking accounts in literature by a fictional psychiatrist, who tells of his assault on a mute, 5-year-old girl. He has chosen her as a victim because she cannot tell anyone of her horrifying experience. The psychiatrist, Bullingdon, recounts:

Those little mad girls, all slack-bodied and soft, great eyes, perfect skin. Examining them... you can imagine. Sometimes they scream. There was this little one with long dark hair, bright eyes, she would not speak, never made a sound, never let anyone touch her, so they brought her to me. She let me touch her. But she was rigid. She knew I wanted to rip her open to fuck her warm little guts.’

Bullingdon then casually notes the result of his treatment of this child: ‘She died. Children die so damned easily’. Such terrifying fictional psychiatrists are likely to be the only representations of the profession that distressed new patients have encountered before meeting a real doctor.

Many novels seem to give good reasons to explain our fear of psychiatry. Beyond rape and murder, fictions also portray psychiatrists as medical torturers. Lobotomy and electroconvulsive therapy (ECT) are shown as devices of control and punishment of the mad in Ken Kesey’s 1962 novel, *One Flew Over the Cuckoo’s Nest*,^11^ later made into a very successful film.^12^ Another quite common fictional theme is the terrifying hospital career of the psychiatric in-patient, and Mary Jane Ward’s 1946 novel, *The Snake Pit*,^13^ is an early and excellent example of this kind of asylum fiction. Ward presents a disturbing account of a patient’s confused experience of a huge state mental hospital, where ECT, lengthy confinement in cold-water baths and packing in icy sheets are treatments ordered by a remote psychiatrist, who also experiments with psychoanalysis. None of the staff explains to the frightened patient, Virginia, what treatment she is to receive or why; or, indeed, that it is treatment intended to help, not punish her. Before ECT, Virginia realises: ‘They were going to electrocute her, not operate upon her... What had you done? You wouldn’t have killed anyone and what other crime is there which exacts so severe a penalty?’

The sins of the fictional psychiatrist are many. They may be cruel jailers, confining patients in frightening places and cutting off access to the world outside, as happens in Antonia White’s *Beyond the Glass*,^14^ Charles Reade’s *Hard Cash*^15^ and Charlotte Brontë’s *Jane Eyre*.^16^ Therapists in fiction sometimes have their own, devious ends in mind when supposedly caring for mad patients: Patrick McGrath’s *Asylum*^17^ is a fine example of this. Still more professionals are frauds and charlatans, such as the amusingly named Beata Pappenheim (a reference to the real name of Breuer’s famous patient, Anna O) in Muriel Spark’s *Aiding and Abetting*.^18^ This wicked group of psychiatric practitioners tricks their patients out of large sums of money, or uses blackmail when privy to hidden information (see, particularly, W. L. Gresham’s *Nightmare Alley*^19^). Italo Svevo’s *Zeno’s Conscience*^20^ is particularly interesting. In this early work, the first psychiatric novel ever published, the treacherous psychiatrist breaks confidentiality by publishing his patient’s private journal. He does this as revenge, thereby proving his untrustworthiness, because his patient has refused to finish his treatment. Psychiatrists in yet another group of novels are shown as racial oppressors, the most well-known of these being Ralph Ellison’s *Invisible Man*;^21^ Jacqueline Roy’s *The Fat Lady Sings*^22^ and Marge Piercy’s *Woman on the Edge of Time*^23^ also describe psychiatry failing ethnic minority patients, either by neglect or by carrying out potentially dangerous surgical experiments. All these fictional psychiatrists are thoroughly nasty people.

## The character of the incompetent psychiatrist

Perhaps less evil, although of no help to their patients, are those fictional practitioners of psychiatry who are just plain useless. Joyce MacIver’s *The Frog Pond*^24^ has a patient who consults a series of ineffectual charlatans. Such fictional ‘doctors’ are often unqualified, incompetent or distractedly focused on their own troubled lives. Philip Roth’s *Portnoy’s Complaint*^25^ entertainingly shows an ineffectual psychiatrist who does not listen to and fails to make contact with his patient. After the reader has worked through some 300 pages of Portnoy’s description of his hilariously troubled life, the psychiatrist, comically named Dr Speilvogel, ends the book with, ‘Now vee may perhaps to begin, yes?’ Roth ridicules this psychiatrist, the doctor’s foreign accent acting as both a reference to Freud and a barrier to communication with his patient. It is noteworthy that the German phrase, ‘Einen Vogel haben’ means ‘to be crazy’, with the implication that we have another mad psychiatrist. While the reader will have picked up many clues to Portnoy’s disturbed state and its origins, the doctor appears to have completely missed all that is relevant to his job as therapist. The reader may imagine Portnoy being thrust even further into his comic despair by this psychiatrist who asks him to start all over again.

## Fictional representations of real psychiatrists

Beyond the invented psychiatrists of novels, real psychiatrists, particularly Freud and Jung, are often fictionalised and are rarely accorded respect. The examples of W. H. Rivers and Freida Fromm-Reichmann, therapists who are both presented as competent, sensitive and caring proponents of the talking cure in Pat Barker’s *Regeneration Trilogy*^26^ and Joanna Greenberg’s *I Never Promised You a Rose Garden*^27^ respectively, are notably unusual. The good, caring psychiatrists in these novels are easily lost among the villains that dominate fiction.

## Looking at the source

What are we to deduce from this great deluge of negative fictional representations? Why do our fictions suggest we are frightened of psychiatrists, fearing they are ‘out to harm us’, manipulate us or defraud us of our money? No other health specialty attracts this hugely negative portrayal.

There are some good reasons for this fear. It is clear that psychiatrists are in the unusual position of having the frightening, legal power to lock up patients. To be classified as mad is to be at the mercy of the psychiatrist-led system, with therapists able to deny patients contact with the outside world and to administer treatments that may well be experienced as punishments for failing to conform to society’s norms of sanity. Historically, psychiatrists have carried out unproven, painful, even permanently damaging treatments on vulnerable patients who no longer have a voice once diagnosed as mad. Some of these ‘cures’ have historical roots in magic or religion only: Michel Foucault, in *Madness and Civilization*,^28^ discusses the origins of water cures in absolution and purification, whereby the patient is offered miraculous rebirth. While many medical specialties have used possibly well-intentioned but nevertheless barbaric ‘cures’ in the past, their modern-day practitioners, unlike psychiatrists, are not held responsible for the errors of their professional forebears.

Fundamentally, psychiatry has always held a strange position in the medical hierarchy: there is no urine test for schizophrenia as there is for diabetes; scientific evidence cannot be presented for bipolar disorder in the way that an oncologist can identify a cancer from a biopsy. The shifting categories of mental illness do not give the general public confidence in psychiatric diagnoses, as the controversial discussions (e.g. Furedi^29^) over the newly published DSM-5^30^ attest. With psychiatry’s position lying between science and art, there still seems to be something of the miracle worker in the role of the psychoanalyst/psychotherapist. I agree with Michel Foucault’s judgement that, after Tuke and Pinel, ‘the psychiatrist’s power [becomes] more and more miraculous, and the doctor-patient couple sinks deeper into a strange world’.^28^ Foucault’s view asserts the peculiarity of psychiatry within the world of medicine, a position which is recognised by many doctors. Psychotherapy and psychoanalysis particularly are curious and hidden processes, involving a lengthy, private relationship between therapist and patient. The terrible fear attached to madness, in which the very means of thinking about reality descend into confusion and chaos, and the perception of the psychiatrist as a miracle worker, ungrounded in accepted medical sciences, confirms, for me, Foucault’s view that the psychiatric patient comes to ‘accept this self-surrender to a doctor both divine and satanic, beyond human measure in any case’.^28^

The significant powers of real psychiatrists and our attitudes towards madness lead us to some understanding of why psychiatry is represented in such a negative way. There are some good reasons for our mistrust and fear of psychiatrists. There is a substantial group within the psychiatric community which is critical of current treatments.^31-35^ Past treatments, such as lobotomy and insulin coma therapy, were often barbaric. Although there has been far-reaching research on the functioning of the brain, our scientific understanding is incomplete. The psychiatrist/patient relationship is different from the usual medical doctor’s dealings with those in need of care, since the psychiatric patient’s very being and perceptions of reality are the matter of treatment. Severe mental distress obviously exists, even though the labels for mental illness constantly change and are determined by mutable ranges of symptoms, rather than by scientific tests. People with mental illness clearly require help to make their lives bearable. To this end, the profession must look seriously at improving its public image, first, by being aware of how damaging the negative fictional portrayals are. These evil fictional psychiatrists are the profession’s emissaries: they represent the psychiatrists most commonly encountered outside hospitals and clinics. Real psychiatrists need to counteract this damaging public image. Further, the profession needs to acknowledge that the significant stigma surrounding mental illness attaches to the doctors as well as the patients. Psychiatrists should pay thoughtful attention to the task of persuading society that they are healers and not torturers, criminals, sexual predators, charlatans and money-grabbing madmen. They must address the way the general public fears and ridicules them. Patients in need of psychiatric care should understand that real psychiatrists are, in general, professionals who are as dedicated to helping those in distress as any doctor is to help his or her patients. If psychiatric practitioners had greater awareness of their appalling, widespread representation in our fictions, it would be a first step to changing the negative views of the psychiatrist with which our culture bombards us. As a literary scholar exploring this field, I urge psychiatrists to work to counteract the harm being caused to the profession by authors of fictions and makers of films. Do not let them be your major ambassadors. Take charge of your public image.
